# Physiological and growth responses to replacing corn gluten with corn fermented protein in diets for juvenile European sea bass: growth performance, intestinal morphology and fillet quality

**DOI:** 10.1007/s10695-026-01710-3

**Published:** 2026-06-03

**Authors:** Ebru Yılmaz, Mehmet Güler, Sema Midilli, Şükrü Yıldırım, Onurkan Antepli, Deniz Çoban

**Affiliations:** 1 Bozdogan Vocational School, Aydın Adnan Menderes University, Aydın, 09760 Türkiye; 2 Faculty of Agriculture, Department of Fisheries Engineering, Aydın Adnan Menderes University, Aydın, 09100 Türkiye; 3 Faculty of Fisheries, Department of Aquaculture, Ege University, Bornova, İzmir Türkiye

**Keywords:** Fish feed, Growth, Juvenile, Intestine histology, Fillet fatty acid profile

## Abstract

This study evaluated the effects of replacing corn gluten with corn fermented protein in the diets of European sea bass (*Dicentrarchus labrax*) juveniles (25.9 ± 0.2 g) during a 60-day feeding trial. Four experimental diets were prepared by replacing 0, 25, 50 and 100% of the corn gluten with corn fermented protein, corresponding to ≈0, 2.25, 4.50 and 9.00% dietary corn fermented protein, all formulated to contain ≈ 47% crude protein and ≈ 18% crude lipid. Growth approximately doubled, with the highest numerical values observed in the 100% replacement group; however, no significant differences were detected among groups in terms of weight gain, daily growth index, feed conversion ratio, or specific growth rate (*p* > 0.05). Hepatosomatic and viscerosomatic index values did not differ significantly among groups. Whole-body proximate composition was generally similar among treatments, although lipid content differed significantly (*p* < 0.05). Although minor numerical variations were observed in fatty acid composition, total saturated fatty acids, monounsaturated fatty acids, polyunsaturated fatty acids, and omega-3, omega-6, and omega-9 contents did not differ significantly among treatments (*p* > 0.05). Hematological and biochemical parameters showed no significant differences between groups, whereas histomorphometric analysis showed higher villus height in the 100% replacement group and increased thickness of selected intestinal layers in corn fermented protein-fed groups. Digestive enzyme activities were significantly affected by dietary treatment and generally increased with corn fermented protein inclusion (*p* < 0.05). Corn fermented protein inclusion numerically reduced diet cost and economic conversion rate. In conclusion, replacing corn gluten with corn fermented protein did not impair growth performance, haematological or serum biochemical parameters, or the whole-body fatty acid profile of juvenile European sea bass under the present experimental conditions, while whole-body lipid content, digestive enzyme activity, and intestinal morphology were affected.

## Introduction

Aquaculture accounts for the vast majority of European sea bass and gilthead sea bream production, and most of this production is concentrated in Mediterranean countries; the main producers are Türkiye and Greece (Zoli et al. 2023). In carnivorous fish farming, feed and feeding costs cover 40 to 70% of operating costs under intensive production conditions (Oliva-Teles et al. 2015). The increase in fish meal prices and the decrease in fish meal availability have led producers and scientists to seek sustainable and economical alternative protein sources (Serra et al. 2024).

Distillers dried grains with solubles (DDGS) are the dry residues obtained from the fermentation of cereal mashes (corn, wheat, sorghum, barley) using specific enzymes and yeasts for ethanol production (Magalhães et al. 2015). In recent years, the inclusion of distillers dried grains with solubles (DDGS), as well as high-protein distillers dried grains (HP-DDG) and corn fermented protein (CFP) by-products, in aquaculture feeds has gained increasing attention within the aquaculture industry. Numerous studies on different marine and freshwater fish species have supported their potential as alternative feed ingredients (Øverland et al. 2013; Tidwell et al. 2017; Goda et al. 2019a,b, 2020; Allam et al. 2020; Barbacariu et al. 2022; Von Eschen et al. 2022; Yıldırım et al. 2024).

The aim of this study was to evaluate the effects of replacing corn gluten with corn fermented protein (CFP) in commercial feed formulations for European sea bass (*Dicentrarchus labrax*). In the present study, corn gluten was selected as the substituted ingredient because it is a commonly used plant protein source in practical sea bass feed formulations, making it a relevant benchmark for evaluating the potential of CFP under commercial diet conditions. In this context, the potential effects of replacing different proportions of CFP on the growth performance and feed utilization efficiency of the fish were examined, and apparent nutrient digestibility was evaluated.

Additionally, distal intestinal histomorphology was analyzed to evaluate the functional integrity of the digestive system, and digestive enzyme activities were measured to assess possible changes in digestive physiology. Furthermore, various hematological parameters were evaluated to provide information on the general physiological status of the fish. Under the conditions tested, these findings may help evaluate whether corn fermented protein can be used in place of corn gluten at the tested inclusion levels without compromising growth performance or physiological status in juvenile European sea bass.

## Material and method

### Fish and experimental facilities

After being transferred to the facility, European sea bass (*Dicentrarchus labrax*) obtained from a private fish farm were released into inlet water with a salinity of 34–35 ppt, treated with UV light, and oxygenated. Then, they were adapted to the experimental conditions by feeding on commercial feed for two weeks before the feeding trials. After the adaptation process, the fish were weighed after a 24-h fasting period at the beginning of the experiment and placed in 12 randomly selected tanks (140 L water capacity) with 16 fish each. Dietary treatments were carried out in three randomly selected tanks for each diet group. The feeding trial lasted a total of 60 days. The fish were hand-fed twice a day (09:00 and 15:00). The amount of feed consumed by the fish in each tank was recorded daily throughout the experiment. At the end of the trial, all of the groups were fasted for 24 h before sampling. Then, total length and weight measurements were taken from all fish.

During the growth trial, the photoperiod was maintained at about 11 h of light and 13 h of darkness. During the trial, water temperature was 22.02 ± 0.08 ^0^C, oxygen saturation was kept above 85%.

Water quality was monitored weekly in the recirculating aquaculture system. As a result of the measurements, it was determined that none of these compounds reached lethal levels for the fish and that the water continued to be protected healthily. Nitrite, nitrate and ammonium concentrations were monitored weekly additionally when required by taking samples from the inlet and outlet of the tanks using a spectrophotometer (Merck Prove 100; Darmstadt, Germany); the mean values of the tank samples were NO₂: 0.16 ± 0.03 ppm, NO₃: 12.5 ± 2.1 ppm, and NH₄: 0.04 ± 0.01 ppm.

### Feed preparation

Experimental diets were prepared at the Aquaculture Research and Application Unit of Aydın Adnan Menderes University, Faculty of Agriculture. Fish meal, fish oil, wheat gluten, corn gluten, soy flour, soy protein concentrate, blood meal, wheat flour, vitamin and mineral premix, BHT, choline C60 and methionine DL99 were supplied by Kılıç Sea Food Co. CFP was supplied by POET Bioproducts (S.A., USA). In terms of amino acid profile, it is especially rich in leucine (6.65%), glutamic acid (7.23%) and valine (2.95%). After the raw nutrient analyses of feed raw materials such as moisture, protein, lipid and ash, the formulation of experimental feeds was prepared to contain similar crude protein (47%) and crude lipid (18%). Additionally, Corn Fermented Protein (CFP) contained 7.0% moisture, 61.1% crude protein, 4.0% crude fat, 2.3% crude ash, and 6.0% crude fiber, with aflatoxin at 2.2 ppb, zearalenone at 24 ppb, and no detectable fumonisin (0.0 ppm) (Table 1).

**Table 1:** Formulation and nutrient content of experimental diets

	Control (%0)	CFP1 (%25)	CFP2 (%50)	CFP3 (%100)
Fish Meal ^a^	32	32	32	32
Fish Oil ^a^	13.30	13.30	13.30	13.30
Wheat Gluten ^a^	7.5	7.5	7.5	7.5
Corn Gluten ^a^	9.00	6.75	4.5	-
Soy Flour ^a^	10.00	10.00	10.00	10.00
Soy Protein Concentrate ^a^	12.00	12.00	12.00	12.00
CFP ^b^	-	2.25	4.5	9.0
Blood Flour ^a^	2.5	2.5	2.5	2.5
Wheat Flour ^a^	11.40	11.40	11.40	11.40
Vitamin Premix ^c^	0.40	0.40	0.40	0.40
Mineral Premix ^d^	0.20	0.20	0.20	0.20
BHT ^a^	0.50	0.50	0.50	0.50
Choline C 60 ^a^	0.20	0.20	0.20	0.20
DL-methionine (DLM, 99%) ^a^	0.50	0.50	0.50	0.50
Cr_2_O_3_^f^	0.50	0.50	0.50	0.50
Total	100	100	100	100
Nutritional Content				
Dry matter (%)	88.26	88.31	88.37	88.47
Crude protein (%)	47.51	47.40	47.29	47.07
Crude lipid (%)	18.03	18.05	18.07	18.11
Crude ash (%)	6.44	6.47	6.50	6.56
NFE (Nitrogen- free extract) (%)	28.02	28.08	28.14	28.26
DE (Digestible energy) (MJ/kg)	2.26	2.25	2.25	2.25
Gross energy (kj/g)	23.18	23.15	23.14	23.12

^a^Fish meal, fish oil, wheat gluten, corn gluten, soy flour, soy protein concentrate, blood meal, wheat flour, Choline C 60 and DL-methionine (DLM, 99%), BHT (butylated hydroxytoluene), Kılıç Sea Food Co., Türkiye. ^b^CFP, POET. ^c^Vitamin mix (mg kg^−1^ feed). ^d^Mineral mix (mg kg^−1^ feed). ^f^Cr_2_O_3_ (chromium (III) oxide). Sigma–Aldrich, St. Louis, MO, USA

Experimental feeds were prepared by adding 0% (Control), 25% (CFP1), 50% (CFP2) and 100% (CFP3) CFP instead of corn gluten, respectively. All raw materials have been screened and passed through a grinder before feed production. The dry ingredients were first mixed, followed by the addition and homogenization of the liquid ingredients in a laboratory-scale feed mixer. The mixing process of the mixture to which water was added was continued and when the appropriate consistency was reached, feeds in the form of 3 mm pellets were prepared. The pellets passed through the La Monferrina—P3 machine were dried in a drying cabinet with air circulation at 40 °C for 10–12 h.

### Feed and fillet analysis

The component analysis of diets and fish fillets was performed as follows: moisture, crude protein, crude ash and crude lipid analyses were determined according to standard methods (AOAC 1998). Two fish per tank were sampled for the fillet analysis. Dry matter content was determined by drying in an oven at 105 °C for 24 h until constant weight was reached. Crude protein was analyzed using the Kjeldahl method (Kjeldahl nitrogen analyzer, model EFLAB MGD-1001). Crude ash was determined by burning in a muffle furnace at 525 °C for 12 h. Crude lipid was extracted with diethyl ether (40–60 °C) and quantified using a Soxhlet extraction device (VELP Scientifica, model SER 148).

### Digestibility analysis

Fecal samples were collected from each tank prior to morning feeding using a feces collection apparatus over a period of 22 days. The collected material was centrifuged at 3000 rpm for 15 min, and the supernatant was removed before storing the samples in sealed Falcon tubes at − 20 °C until analysis (Diogenes et al. 2018). The proximate composition of feces was analyzed for moisture, crude protein, crude ash, and crude lipid following standard AOAC procedures (1998). Briefly, dry matter was determined by drying samples in an oven at 105 °C until a constant weight; ash content was measured after incineration in a muffle furnace at 525 °C for 12 h; crude protein (N × 6.25) was quantified using the Kjeldahl method following acid digestion with Kjeltec digestion and distillation units; and crude lipid was extracted with diethyl ether using a Soxtec HT System. Chromium oxide concentrations in diets and feces were determined by the acid digestion method of Furukawa and Tsukahara (1966). Apparent digestibility coefficients (ADCs) for dry matter, protein, and lipid were then calculated using the following formula:

$$ADC\left(\%\right)=100-\left[100\times \left({Cr}_{2}{O}_{3 }\,in \,diet/{Cr}_{2}{O}_{3 }in\, feces\right)\times \left(nutrient\, in\, feces/nutrient \,in \,diet\right)\right]$$

### Growth indices

At the beginning and end of the experiment, total length and weight of all fish were measured. Fish were anesthetized with herbal anaesthetic (AquaSed 20 mg L⁻^1^, https://www.nativital.com/) considering animal welfare. Growth performance and feed utilization were evaluated using the following formulas (IBW: initial body weight; FBW: final body weight):

$$\begin{array}{c}\begin{array}{cc}WG\,\left(Weight\,gain\%\right)&=100\times\left(FBW-IBW\right)/IBW\end{array}\\\begin{array}{cc}FCR\,\left(Feed\,conversion\,ratio\right)&=Feed\,intake/Weight\,gain\end{array}\\\begin{array}{c}\begin{array}{cc}DGI\left(Daily\,growth\,index\right)&=100\times\left[\left({FBW}^{\left(1/3\right)}-{IBW}^{\left(1/3\right)}\right)/days\right]\end{array}\\\begin{array}{cc}SGR\,\left(Specific\,growth\,rate;\%/day\right)&=100\times\left[ln\left(FBW\right)-ln\left(IBW\right)\right]/days\end{array}\\\begin{array}{c}\begin{array}{cc}Survival\left(\%\right)&=100\times\left(Number\,of\,survivors/Initial\,number\right)\end{array}\\\begin{array}{cc}HSI\left(Hepatosomatic\,index\right)&=100\times\left(Liver\,weight/Body\,weight\right)\end{array}\\\begin{array}{cc}VSI\left(Viscerosomaticindex\right)&=100\times\left(Viscera\,weight/Body\,weight\right)\end{array}\end{array}\end{array}\end{array}$$

### Determination of fatty acids in fish fillets

Total lipids were extracted following the method of Bligh and Dyer (1959). The fatty acid composition of the samples was analyzed using a Thermo Scientific ISQ LT GC/MS system equipped with a 60 m Trace Gold TG-WaxMS capillary column (Thermo Scientific, code 26088–1540). The column featured an internal diameter of 0.25 mm and a film thickness of 0.25 μm. The injector temperature was set at 240 °C, while the oven temperature was initially maintained at 100 °C for 3 min and subsequently increased to 240 °C at a rate of 4 °C per minute. Helium was used as the carrier gas at a constant flow rate of 1 ml/min with a split ratio of 1:20. The MS detector (ISQ LT) operated in electron ionization mode at 70 eV. Fatty acids were identified by comparing their retention times with those of a 37-component FAME standard mixture (Çorapci et al. 2021).

Total fatty acid groups were calculated using the following formulas;

$$\begin{aligned}&\sum SFA=C4{\mathrm{:}}0+C6{\mathrm{:}}0+C8{\mathrm{:}}0+C10{\mathrm{:}}0+C11{\mathrm{:}}0+C12{\mathrm{:}}0+C13{\mathrm{:}}0+C14{\mathrm{:}}0+C15{\mathrm{:}}0+C16{\mathrm{:}}0+C17{\mathrm{:}}0+C18{\mathrm{:}}0+C20{\mathrm{:}}0+C21{\mathrm{:}}0+C22{\mathrm{:}}0+C23{\mathrm{:}}0+C24{\mathrm{:}}0\\&\sum MUFA=C14{\mathrm{:}}1+C15{\mathrm{:}}1+C16{\mathrm{:}}1+C17{\mathrm{:}}1+C18{\mathrm{:}}1n{\mathrm{-}}9c+C18{\mathrm{:}}1n{\mathrm{-}}9t+C20{\mathrm{:}}1n{\mathrm{-}}9c+C22{\mathrm{:}}1n{\mathrm{-}}9+C24{\mathrm{:}}1\\&\sum PUFA=C18{\mathrm{:}}2n6t+C18{\mathrm{:}}2n6c+C18{\mathrm{:}}3n3+C18{\mathrm{:}}3n6+C20{\mathrm{:}}2+C22{\mathrm{:}}2+C20{\mathrm{:}}3n3+C20{\mathrm{:}}3n6+C20{\mathrm{:}}5n{\mathrm{-}}3+C20{\mathrm{:}}4n{\mathrm{-}}6+C22{\mathrm{:}}6n{\mathrm{-}}3\\&\sum Omega{\mathrm{-}}3(\omega3)=C18{\mathrm{:}}3n{\mathrm{-}}3+C20{\mathrm{:}}3n{\mathrm{-}}3+C20{\mathrm{:}}5n{\mathrm{-}}3+C22{\mathrm{:}}6n{\mathrm{-}}3\\&\sum Omega{\mathrm{-}}6(\omega6)=C18{\mathrm{:}}2n{\mathrm{-}}6t+C18{\mathrm{:}}2n{\mathrm{-}}6c+C18{\mathrm{:}}3n{\mathrm{-}}6+C20{\mathrm{:}}4n{\mathrm{-}}6+C20{\mathrm{:}}3n{\mathrm{-}}6\\&\sum Omega{\mathrm{-}}9(\omega9)=C18{\mathrm{:}}1n{\mathrm{-}}9c+C18{\mathrm{:}}1n{\mathrm{-}}9t+C20{\mathrm{:}}1n{\mathrm{-}}9c+C22{\mathrm{:}}1n{\mathrm{-}}9\end{aligned}$$

### Blood analysis

Blood samples were obtained from available sampled fish within each tank, with one to three measurements available per tank. Haematological and serum biochemical values were averaged within each tank to obtain one tank-level value before statistical analysis. The fish were fasted for 1 day before blood samples were taken. After being randomly captured and swiftly removed from the tanks, the fish were anesthetized with a 20 mg L⁻^1^ herbal anesthetic (AquaSed) used as an analgesic. After anaesthesia, the area immediately behind the anal fin of the fish was cleaned with alcohol (to prevent contamination of the blood with mucus) and then a blood sample was taken from the caudal vein with a 2.5 ml plastic syringe. Blood samples were separated into K3EDTA and gel serum tubes and haematological and serum biochemical analyses were performed. For serum analysis, blood samples taken into gel tubes were centrifuged at 5000 x g for 10 min. The serum obtained was stored at −80 °C until analysed (Yılmaz and Ergün, 2018).

#### Haematological analyses

Haematological parameters were evaluated using a haematological analyzer (EDAN-H30 VET) (Ahmad and Lashari 2022).

#### Biochemical analyses

In the experiment, total protein (TP) (Catalog no: OttoBC154), albumin (ALB) (Catalog no: OttoBC123), globulin (GLO) (Catalog no: OttoBC142), triglyceride (TG) (Catalog no: OttoBC155), very low density lipoprotein (VLDL), cholesterol (CHOL) (Catalog no: OttoBC135), high density lipoprotein (HDL) (Catalog no: OttoBC144), low density lipoprotein (LDL) (Catalog no: OttoBC145), creatinine (CRE) (Catalog no: OttoBC139), uric acid (UA) (Catalog no: OttoBC157) levels were measured by colorimetric method (fully automatic biochemistry device, Mindray-BS400) (Cetik Yildiz et al. 2024; Caglar et al. 2024).

### Determination of colour in diet, scale and fillets

At the end of the experiment, the feed, fillets and scale colours of experimental fish fed with control and CFP were measured using a colour spectrophotometer (ColorFlex EZ, HunterLab, USA). The definitions of L ∗, a ∗ and b ∗ parameters are as follows: L* brightness (light–dark), a* (redness-greenness), b* (yellowness-blueness). Three repeated measurements were taken for each sample (Hunter 1975).

### Histological analysis

For histological examination, samples were collected from five fish per tank at the end of the experiment. The samples were a piece of the distal intestine and liver tissue. The tissues was fixed formalin solution, then processed by alcohol series and xylene and embedded in paraffin, and consequently cut into 5 µm thickness. These tissues were put on slides and stained with the hematoxylin and eosin method (Luna 1968). In histological measurement, villus height, villus width, lamina propria, submucosa, stratum compactum, circular muscularis, and muscularis parameters were determined and measured as to prior studies (Krogdahl et al. 2003; Ye et al. 2016; Bonvini et al. 2018; Aydın and Gümüş, 2020). Stained slides were examined under a light microscope (Olympus CX31, Olympus Inc., Japan), measurements were performed using LABSENS software, and microphotographs were taken with a camera attached to the microscope (Olympus DP20). Any morphological differences concerning enteritis in the distal intestine (Krogdahl et al. 2003; Ye et al. 2016; Bonvini et al. 2018; Goda et al. 2020; Kumar et al. 2020) and lipid accumulation in the liver were evaluated as to previous studies (Caballero et al. 2004; Goda et al. 2020; Robaina et al. 1998).

### Enzymatic analysis

At the beginning of the experiment, three fish were sampled, whereas at the end of the experiment, one fish from each tank was sampled for enzyme analysis.

#### Gastrointestinal dissection

Isolation process of the gastrointestinal segment required for enzyme assays were performed as described by Yıldırım et al. (2024). After homogenization of dissected segments were stored −20 °C with addition of purified water, glycerol (Isolab) and Tris HCl (Sigma-Aldrich) with a pH value of 7.5. Each enzyme was identified using specific determination methods, and enzyme activities were quantified using by Jenway 6300UV-Vis spectrophotometer.

#### Analytical procedure

Alkaline protease activity was determined using casein as the substrate, following the method of Alarcón et al. (1998). Enzyme activity was calculated from spectrophotometric readings at 366 nm under the assay conditions of 37 °C and pH 8.0. Acid protease activity was assayed using bovine hemoglobin as the substrate, with absorbance measured at 280 nm after incubation for 30 min at 25 °C (Anson 1938). Amylase activity was evaluated according to the method of Métais and Bieth (1968), using starch as the substrate and recording absorbance at 540 nm over a 5-min reaction period. Lipase activity was determined according to the method of McKellar and Cholette (1986), as modified by Versaw et al. (1989), using β-naphthyl caprylate as the substrate. One unit of enzyme activity was defined as the amount of lipase releasing 1 mg of β-naphthol per minute, measured at 490 nm during a 10-min reaction period. All enzyme activities were expressed as specific activity (U/mg protein), and protein concentrations were determined by the Bradford method (Bradford 1976).

### Economic analysis

The economic value of the diets was determined by the following calculations (Abdel Rahman et al. 2010; Salama et al. 2010).

$$Economic \,conversion \,rate\left(ECR\right)=FCR\times \,Cost\, of \,diet\, \left(\$/kg\right)$$

### Statistical analysis

Data are presented as mean ± standard error (SE), unless otherwise stated. The analytical unit and the n used for statistical analysis are indicated in the corresponding tables and figure legends. Statistical analyses were performed using SPSS software, and statistical significance was accepted at *p* < 0.05. Normality and homogeneity of variance were assessed using the Shapiro–Wilk and Levene’s tests, respectively.

For variables related to growth performance, organ indices, whole-body proximate composition, haematological parameters, serum biochemical parameters, and final digestive enzyme activities, measurements obtained within each tank were averaged where applicable to obtain one tank-level value. These tank-level values were used for group comparisons (n = 3 per dietary group). These variables were analysed primarily by one-way ANOVA. Tukey’s HSD test was used for post hoc comparisons when appropriate. Because of the limited number of tank replicates, Kruskal–Wallis tests were additionally performed as sensitivity analyses.

For fatty acid composition, one fish per tank was analysed and three technical measurements per sample were averaged to obtain one tank-level value (n = 3 per dietary group). Fatty acid composition, scale and fillet colour, and digestibility variables were analysed using the Kruskal–Wallis test because normality assumptions were not consistently met and group sizes were small. When applicable, Dunn–Bonferroni pairwise comparisons were used following significant Kruskal–Wallis results. Feed colour was based on three technical readings per diet and was presented descriptively without inferential statistical testing. Hepatocyte diameter and intestinal histomorphometric parameters were analysed using the Kruskal–Wallis test followed by Dunn–Bonferroni pairwise comparisons where appropriate. These data are reported as histological measurements, and the number of measurements used for each parameter is stated in the corresponding table notes.

As a complementary exploratory analysis, Pearson correlation coefficients were calculated among selected haematological, serum biochemical, and digestive enzyme variables using tank-level values (n = 12). Variables directly derived from other measured parameters, such as GLB and VLDL, were not included in the heat map to avoid formula-driven correlations. Because of the limited sample size and physiological or analytical relationships among some variables, correlations were interpreted descriptively.

## Results

### Growth performance

European sea bass consumed all the feeds given during the trial period. Fish body weight nearly doubled during the trial period. The CFP3 group showed slightly higher numerical growth values compared to the control and other experimental groups; however, no significant differences were detected in growth performance or feed utilization indices (FBW, WG, DGI, FCR and SGR) among dietary treatments based on both parametric (ANOVA) and non-parametric (Kruskal–Wallis) analyses (*p* > 0.05) (Table 2).

**Table 2:** Growth parameters of sea bass fed with different experimental diets CFP (0%, 25%, 50%, and 100%) for 60 days

	CFP supplementation level (%)			
	Control	CFP1	CFP2	CFP3
IBW (g)	25.9 ± 0.2	25.9 ± 0.2	25.9 ± 0.2	25.9 ± 0.2
FBW (g)	48.4 ± 2.0	50.2 ± 1.0	51.1 ± 0.9	52.1 ± 0.1
WG (%)	86.7 ± 7.8	93.8 ± 3.7	97.1 ± 3.4	100.8 ± 0.4
DGI	1.14 ± 0.08	1.21 ± 0.04	1.25 ± 0.04	1.29 ± 0.01
FCR	1.50 ± 0.16	1.31 ± 0.04	1.26 ± 0.04	1.20 ± 0.0
SGR (%/day)	1.04 ± 0.07	1.10 ± 0.03	1.13 ± 0.03	1.16 ± 0.0
Survival Rate (%)	98	98	98	100

Values are mean ± SE of tank mean values (*n* = 3 per dietary group). No significant differences were detected among treatments (*p* > 0.05). Abbreviations: *IBW* initial body weight, *FBW* final body weight, *WG* weight gain, *DGI* daily growth index, *FCR* feed conversion ratio, *SGR* specific growth rate

### Internal organ index

**Fig. 1:**
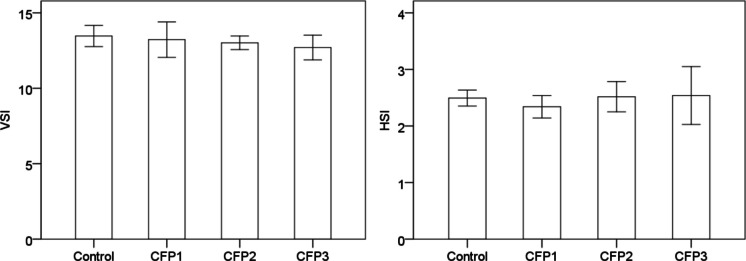
Viscerosomatic index (VSI) and hepatosomatic index (HSI) of sea bass fed diets with increasing CFP inclusion levels. Values are presented as tank-level means (n = 3 per dietary group), and error bars represent 95% confidence intervals. No significant differences were detected among dietary groups for VSI or HSI (*p* > 0.05)

HSI and VSI values did not differ significantly among dietary groups when analyzed using tank-level means (*p* > 0.05). Although VSI was numerically lowest in the CFP3 group, this difference was not statistically significant (Fig. 1).

### Chemical nutrient composition of fish fillets

Whole-body proximate composition was generally similar among dietary treatments, with no significant differences observed in moisture, ash, or protein contents according to both ANOVA and Kruskal–Wallis tests. However, lipid content increased with CFP inclusion and differed significantly among groups (*p* < 0.05) (Table 3).

**Table 3:** Whole-body proximate composition of sea bass fed with different experimental diets CFP (0%, 25%, 50%, and 100%) for 60 days

	CFP supplementation level (%)			
	Control	CFP1	CFP2	CFP3
Moisture	75.05 ± 0.24	75.26 ± 0.17	75.38 ± 0.12	75.19 ± 0.21
Ash	1.29 ± 0.04	1.35 ± 0.00	1.36 ± 0.01	1.35 ± 0.01
Protein	19.94 ± 0.20	19.70 ± 0.22	19.77 ± 0.39	19.81 ± 0.23
Lipid	2.38 ± 0.01^a^	2.42 ± 0.02^ab^	2.46 ± 0.02^b^	2.53 ± 0.01^c^

Values are mean ± SE of tank mean values (*n* = 3 per dietary group). Different superscript letters in the lipid row indicate significant differences among treatments (*p* < 0.05)

### Fatty acid composition of fish fillets

**Fig. 2:**
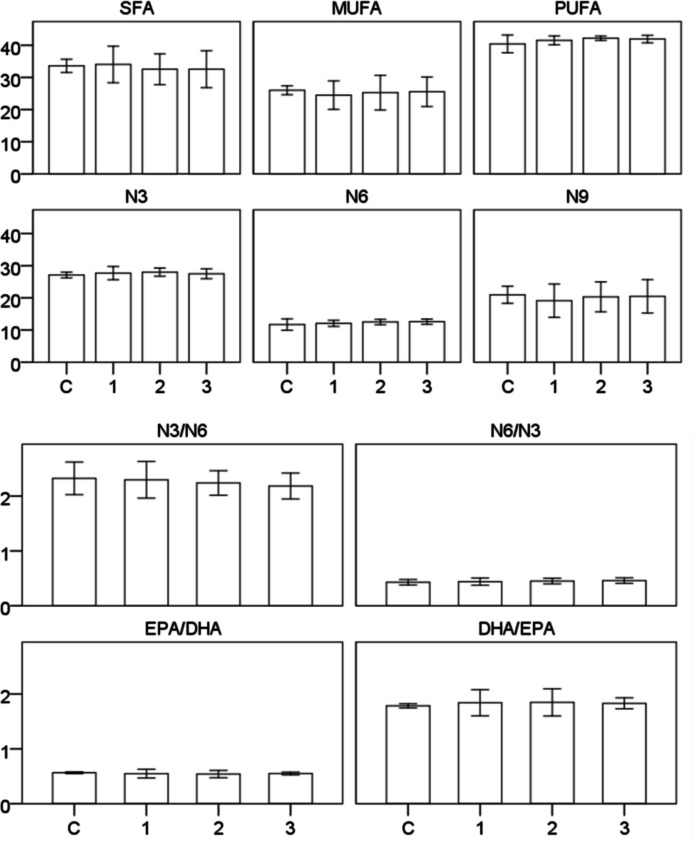
Whole-body fatty acid composition (ΣSFA, ΣMUFA, ΣPUFA, Σn3, Σn6, Σn9, n3/n6, n6/n3, EPA/DHA, and DHA/EPA) of European sea bass fed experimental diets containing 0% (C), 25% (1), 50% (2), and 100% (3) CFP for 60 days. Differences among dietary groups were analyzed using the Kruskal–Wallis test; no significant differences were detected among dietary groups (*p* > 0.05). Error bars represent 95% confidence intervals, n = 3 per dietary group

Whole-body fatty acid composition was generally similar among dietary groups (Table 4). No significant differences were detected among treatments for the major fatty acid groups, including total saturated fatty acids (ΣSFA), monounsaturated fatty acids (ΣMUFA), polyunsaturated fatty acids (ΣPUFA), n-3, n-6, and n-9 fatty acids (*p* > 0.05). Although ΣPUFA values were numerically higher in the CFP-fed groups than in the control group, this difference was not statistically significant. Similarly, the n-3/n-6 and n-6/n-3 ratios, as well as the EPA/DHA and DHA/EPA ratios, did not differ significantly among treatments (*p* > 0.05). Overall, replacing corn gluten with CFP did not significantly alter the whole-body fatty acid profile of European sea bass under the present experimental conditions (Table 4; Fig. 2).

**Table 4:** Whole-body fatty acid composition of sea bass fed with different experimental diets (0%, 25%, 50%, and 100%) for 60 days

Fatty acid	Control	CFP1	CFP2	CFP3
C4:0	0.00 ± 0.00	0.00 ± 0.00	0.00 ± 0.00	0.00 ± 0.00
C6:0	0.01 ± 0.01	0.00 ± 0.00	0.00 ± 0.00	0.00 ± 0.00
C8:0	0.00 ± 0.00	0.00 ± 0.00	0.00 ± 0.00	0.00 ± 0.00
C10:0	0.02 ± 0.00	0.01 ± 0.01	0.02 ± 0.00	0.01 ± 0.00
C11:0	0.00 ± 0.00	0.00 ± 0.00	0.00 ± 0.00	0.00 ± 0.00
C12:0	0.09 ± 0.00	0.09 ± 0.01	0.08 ± 0.01	0.08 ± 0.01
C13:0	0.09 ± 0.00	0.09 ± 0.01	0.07 ± 0.00	0.07 ± 0.01
C14:0	5.30 ± 0.09	5.36 ± 0.07	5.05 ± 0.04	5.14 ± 0.11
C14:1	0.69 ± 0.02	0.70 ± 0.06	0.63 ± 0.04	0.60 ± 0.05
C15:0	1.59 ± 0.04	1.65 ± 0.10	1.49 ± 0.05	1.44 ± 0.10
C15:1	0.36 ± 0.01	0.37 ± 0.03	0.34 ± 0.03	0.32 ± 0.03
C16:0	14.96 ± 0.26	15.15 ± 0.68	14.55 ± 0.61	14.61 ± 0.85
C16:1	1.76 ± 0.03	1.83 ± 0.08	1.53 ± 0.15	1.66 ± 0.08
C17:0	1.71 ± 0.01	1.75 ± 0.05	1.72 ± 0.03	1.68 ± 0.05
C17:1	1.31 ± 0.01	1.39 ± 0.09	1.34 ± 0.07	1.30 ± 0.02
C18:0	6.68 ± 0.10	6.92 ± 0.34	6.64 ± 0.16	6.73 ± 0.22
C18:1n9t	1.41 ± 0.03	1.42 ± 0.22	1.60 ± 0.17	1.69 ± 0.18
C18:1n9c	17.80 ± 0.30	16.61 ± 1.18	17.53 ± 1.04	17.83 ± 1.23
C18:2n6c	7.15 ± 0.23	7.34 ± 0.20	7.71 ± 0.12	7.73 ± 0.11
C18:2n6t	0.97 ± 0.01	0.97 ± 0.02	0.97 ± 0.04	0.96 ± 0.02
C18:3n3	2.55 ± 0.02	2.57 ± 0.02	2.54 ± 0.03	2.50 ± 0.03
C18:3n6	0.43 ± 0.02	0.42 ± 0.01	0.42 ± 0.02	0.45 ± 0.01
C20:0	1.62 ± 0.01	1.69 ± 0.07	1.62 ± 0.05	1.59 ± 0.04
C20:1n9c	1.03 ± 0.46	0.59 ± 0.00	0.60 ± 0.02	0.60 ± 0.01
C20:2	1.49 ± 0.02	1.59 ± 0.03	1.54 ± 0.02	1.59 ± 0.05
C20:3n3	2.40 ± 0.05	2.51 ± 0.06	2.40 ± 0.08	2.34 ± 0.06
C20:3n6	0.44 ± 0.22	0.65 ± 0.02	0.65 ± 0.02	0.66 ± 0.01
C20:4n6	2.71 ± 0.02	2.70 ± 0.04	2.74 ± 0.03	2.80 ± 0.06
C20:5n3—EPA	7.96 ± 0.04	7.96 ± 0.03	8.09 ± 0.08	8.00 ± 0.08
C21:0	0.04 ± 0.00	0.04 ± 0.00	0.04 ± 0.01	0.04 ± 0.00
C22:0	0.44 ± 0.18	0.23 ± 0.20	0.22 ± 0.20	0.09 ± 0.01
C22:2	0.12 ± 0.05	0.15 ± 0.06	0.17 ± 0.06	0.23 ± 0.01
C22:6n3—DHA	14.19 ± 0.13	14.65 ± 0.50	14.95 ± 0.37	14.65 ± 0.31
C22:1n9	0.71 ± 0.04	0.51 ± 0.17	0.58 ± 0.20	0.36 ± 0.19
C23:0	0.14 ± 0.02	0.17 ± 0.03	0.12 ± 0.01	0.12 ± 0.02
C24:0	0.90 ± 0.01	0.86 ± 0.03	0.92 ± 0.01	0.95 ± 0.04
C24:1	0.93 ± 0.25	1.10 ± 0.14	1.11 ± 0.15	1.16 ± 0.02
ΣSFA	33.60 ± 0.48	34.02 ± 1.32	32.54 ± 1.11	32.55 ± 1.33
ΣMUFA	26.00 ± 0.32	24.50 ± 1.03	25.25 ± 1.25	25.52 ± 1.07
ΣPUFA	40.42 ± 0.64	41.52 ± 0.32	42.20 ± 0.16	41.92 ± 0.27
Σn3	27.10 ± 0.21	27.70 ± 0.48	27.98 ± 0.30	27.49 ± 0.36
Σn6	11.70 ± 0.41	12.08 ± 0.22	12.50 ± 0.19	12.61 ± 0.19
Σn9	20.96 ± 0.61	19.12 ± 1.20	20.31 ± 1.08	20.48 ± 1.21
n3/n6	2.32 ± 0.07	2.30 ± 0.08	2.24 ± 0.05	2.18 ± 0.05
n6/n3	0.43 ± 0.01	0.44 ± 0.02	0.45 ± 0.01	0.46 ± 0.01
EPA/DHA	0.56 ± 0.00	0.55 ± 0.02	0.54 ± 0.02	0.55 ± 0.01
DHA/EPA	1.78 ± 0.01	1.84 ± 0.06	1.85 ± 0.06	1.83 ± 0.02

Values are expressed as percentage of total fatty acids and presented as mean ± SE of tank mean values (*n* = 3 per dietary group). Differences among dietary groups were analyzed using the Kruskal–Wallis test; no significant differences were detected among dietary groups (*p* > 0.05)

### Hematological and serum biochemistry

No significant differences were detected among dietary treatments in white blood cells, red blood cells, haemoglobin, or haematocrit values (*p* > 0.05) (Table 5).

**Table 5:** Haematological parameters of sea bass fed with different experimental diets CFP (0%, 25%, 50%, and 100%) for 60 days

	CFP supplementation level (%)			
	Control	CFP1	CFP2	CFP3
WBCs (× 10^9^/L)	0.92 ± 0.54	2.45 ± 1.55	1.97 ± 0.84	1.30 ± 0.37
RBCs (× 10^12^/L)	0.97 ± 0.49	1.53 ± 0.16	1.82 ± 0.18	1.78 ± 0.17
Hb (g/L)	24.50 ± 12.05	36.78 ± 2.39	47.00 ± 5.13	44.33 ± 3.09
Hct (%)	12.07 ± 6.67	20.84 ± 0.90	25.03 ± 2.93	26.45 ± 3.06

Values are mean ± SE of tank mean values (*n* = 3 per dietary group). For each tank, available haematological measurements were averaged prior to statistical analysis. No significant differences were detected among dietary treatments (*p* > 0.05). Abbreviations: *WBC* white blood cells, *RBC* red blood cells, *Hb* haemoglobin, *Hct* haematocrit

CFP supplementation did not cause any significant change on total protein, albumin, globulin, triglyceride, cholesterol, HDL, LDL, VLDL, uric acid and creatinine levels in experimental fish (*p* > 0.05) (Table 6).

**Table 6:** Biochemical parameters of sea bass fed with different experimental diets CFP (0%, 25%, 50%, and 100%) for 60 days

	CFP supplementation level (%)			
Param	Control	CFP1	CFP2	CFP3
TP (g/dL)	6.01 ± 0.09	6.58 ± 0.10	6.24 ± 0.30	6.60 ± 0.20
ALB (g/dL)	1.45 ± 0.03	1.54 ± 0.01	1.49 ± 0.04	1.55 ± 0.06
GLB (g/dL)	4.57 ± 0.07	5.03 ± 0.09	4.75 ± 0.25	5.05 ± 0.15
TG (mg/dL)	262.00 ± 40.36	319.50 ± 20.94	255.17 ± 43.42	326.67 ± 7.55
CHOL (mg/dL)	222.33 ± 17.03	252.44 ± 5.15	223.67 ± 22.45	263.33 ± 7.07
HDL (mg/dL)	93.17 ± 13.98	90.16 ± 8.61	104.37 ± 4.16	98.98 ± 4.46
LDL (mg/dL)	17.50 ± 2.75	19.61 ± 1.07	19.67 ± 4.69	21.00 ± 0.87
VLDL (mg/dL)	52.40 ± 8.07	63.90 ± 4.19	51.03 ± 8.68	65.33 ± 1.51
UA (mg/dL)	0.54 ± 0.12	0.24 ± 0.04	0.37 ± 0.19	0.28 ± 0.06
CREA (mg/dL)	0.43 ± 0.07	0.51 ± 0.05	0.37 ± 0.04	0.49 ± 0.03

Values are mean ± SE of tank mean values (*n* = 3 per dietary group); measurements were averaged prior to statistical analysis. No significant differences were detected among dietary treatments for any serum biochemical parameter (*p* > 0.05). Abbreviations: *TP* total protein, *ALB* albumin, *GLB* globulin, *TG* triglycerides, *CHOL* cholesterol, *HDL* high-density lipoprotein, *LDL* low-density lipoprotein, *VLDL* very-low-density lipoprotein, *UA* uric acid, *CREA* creatinine

### Color analysis of fish feed, scale and fillets

Diet colour values differed descriptively among experimental diets (Table 7), with CFP-containing diets generally showing higher L*, a*, and b* values than the control diet. Because feed colour values were based on technical measurements per diet, they were not subjected to inferential statistical testing. No significant differences were detected among dietary groups for scale colour parameters (L*, a*, and b*) or fillet colour parameters (L*, a*, and b*) (*p* > 0.05). These results indicate that the descriptive colour differences observed in the diets were not reflected in scale or fillet colour after the 60-day feeding trial.

**Table 7:** L*, a* and b* values of feed, scale and fillet of sea bass fed with different experimental diets (0%, 25%, 50% and 100%) for 60 days

	CFP supplementation level (%)			
Feed	Control	CFP1	CFP2	CFP3
L*	4.85 ± 0.00	6.57 ± 0.00	6.77 ± 0.01	5.63 ± 0.01
a*	3.10 ± 0.03	6.08 ± 0.04	6.35 ± 0.02	5.09 ± 0.02
b*	4.91 ± 0.06	8.57 ± 0.00	9.05 ± 0.02	7.30 ± 0.03
Scale	Control	CFP1	CFP2	CFP3
L*	18.73 ± 1.99	15.89 ± 2.38	19.55 ± 2.95	23.86 ± 5.21
a*	0.42 ± 0.26	0.43 ± 0.17	0.31 ± 0.43	0.24 ± 0.12
b*	6.89 ± 0.74	6.99 ± 0.37	7.81 ± 1.11	8.29 ± 1.04
Fillet	Control	CFP1	CFP2	CFP3
L*	35.53 ± 3.44	29.06 ± 1.53	35.72 ± 2.95	38.15 ± 1.59
a*	−1.67 ± 0.71	−1.33 ± 0.58	0.90 ± 1.09	0.73 ± 1.12
b*	12.80 ± 0.71	11.00 ± 0.29	13.90 ± 2.01	14.69 ± 0.71

Values are presented as mean ± SE. Feed colour values are descriptive means of three technical measurements per diet. Scale and fillet colour values are presented as mean ± SE of tank mean values (*n* = 3 per dietary group). No significant differences were detected among dietary groups for scale or fillet colour parameters (*p* > 0.05). Abbreviations: *L* brightness (lightness-darkness), *a* reddish-greenish, *b* yellowish-blue

### Digestibility

Dry matter apparent digestibility coefficients (ADC) varied between 79 and 83%, protein ADCs varied between 92 and 93%, and lipid ADCs varied between 89 and 92%, and no statistically significant difference was found among the groups (*p* > 0.05) (Table 8).

**Table 8:** Digestibility values of sea bass fed with different experimental diets (0%, 25%, 50%, and 100%) for 60 days

	CFP supplementation level (%)			
	Control	CFP1	CFP2	CFP3
Dry matter	79.99 ± 0.62	81.97 ± 0.94	82.17 ± 2.55	83.79 ± 1.47
Protein	92.59 ± 0.43	93.25 ± 0.08	92.93 ± 0.61	93.06 ± 0.66
Lipid	89.83 ± 0.97	91.99 ± 0.91	92.39 ± 0.84	90.58 ± 3.14

Values are mean ± SE of tank-level values (*n* = 3 per group). No significant differences were detected among dietary groups (Kruskal–Wallis test, *p* > 0.05)

### Histological

#### Liver

**Fig. 3:**
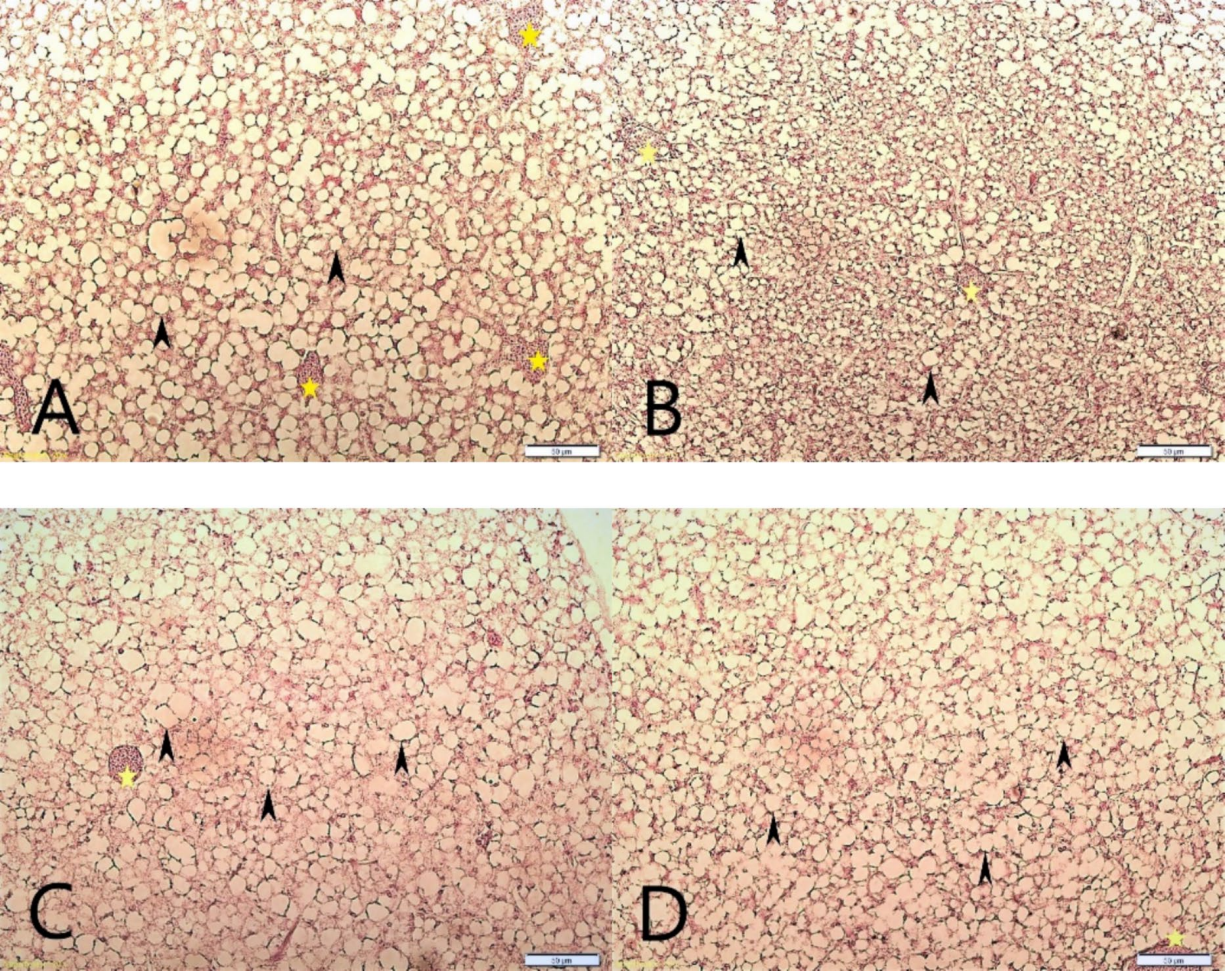
General view of hepatocytes. **A**. Control group 200x, **B**. CFP1 group 200x, **C**. CFP2 group 200x, **D**. CFP3 group 200x, arrow heads: cytoplasmic vacuolation in hepatocyte, stars: congestion in the blood capillaries

Hepatocytes showed cytoplasmic vacuolization, and the nucleus replaced to the peripheral position in all groups. Measured hepatocyte diameter values differed between the groups (*p* < 0.05) (Table 9). Hepatocyte cell diameters of the CFP2 and CFP3 groups were measured to be higher than in the control and CFP1 groups. No melanomacrophage centers which a sign of pathological effect, were observed in any study groups (Fig. 3).

**Table 9:** Hepatic cell diameters

Groups	Hepatocyte cell diameter (µm)
Control	10.76 ± 0.21^a^
CFP1	9.42 ± 0.34^a^
CFP2	13.63 ± 0.55^b^
CFP3	12.91 ± 0.29^b^

Values are mean ± SE of histological measurements (n = 30 measurements per dietary group). Different superscript letters indicate significant differences based on Dunn–Bonferroni pairwise comparisons following the Kruskal–Wallis test

### Distal intestine

**Fig. 4:**
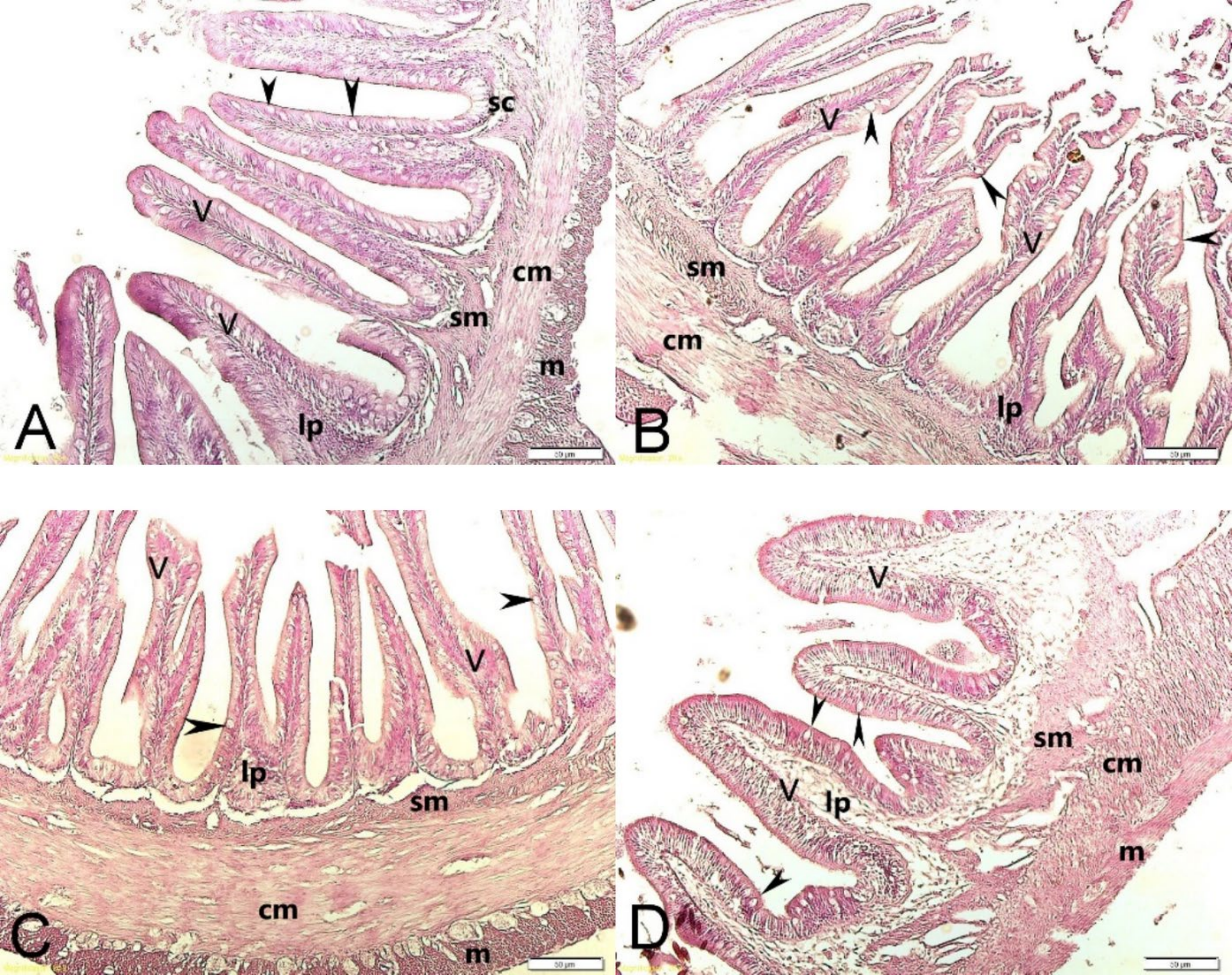
Histomorphology of the distal intestine. A. Control group 200x, B. CFP1 group 200x, C. CFP2 group 200x, D. CFP3 group 200x, v: villus, lp: lamina propria, sc: stratum compactum, sm: submucosa, cm: circular muscularis, m: muscularis; arrow head: goblet cells

In the distal intestine, villus height, submucosa, stratum compactum, and muscularis differed significantly among groups (*p* < 0.05), whereas villus width, lamina propria, and circular muscularis did not differ (*p* > 0.05) (Table 10). Villus height was highest in the CFP3 group. Submucosa was lower in the Control group than in all CFP-supplemented groups. Stratum compactum was lower in the Control group than in CFP2 and CFP3, while CFP1 showed intermediate values. Muscularis thickness was highest in the CFP3 group and differed from the Control group. Histological views of the distal intestine are shown in Fig. 4.

**Table 10:** Histomorphometric parameters of intestine

Parameters	Control	CFP1	CFP2	CFP3
Villus height	564.0 ± 15.6^a^	560.3 ± 11.7^a^	581.7 ± 8.6^ab^	602.1 ± 8.9^b^
Villus width	85.0 ± 3.1	80.8 ± 3.5	86.9 ± 2.4	90.5 ± 2.0
Lamina propria	6.86 ± 0.33	6.57 ± 0.38	6.97 ± 0.35	7.17 ± 0.24
Submucosa	20.25 ± 0.69^a^	23.94 ± 0.44^b^	25.05 ± 0.50^b^	25.53 ± 0.59^b^
Stratum compactum	5.26 ± 0.16^a^	5.64 ± 0.21^ab^	6.18 ± 0.17^b^	6.59 ± 0.32^b^
Circular muscularis	51.72 ± 1.27	53.93 ± 1.10	54.13 ± 1.36	55.48 ± 1.58
Muscularis	26.05 ± 0.83^a^	27.38 ± 0.94^ab^	28.22 ± 0.88^ab^	30.89 ± 1.19^b^

Values are presented as mean ± SE of histological measurements. All measurements are expressed in µm. Sample sizes were *n* = 33, 30, 30, and 30 for villus height and villus width measurements in the Control, CFP1, CFP2, and CFP3 groups, respectively, and *n* = 20 per group for lamina propria, submucosa, stratum compactum, circular muscularis, and muscularis measurements. Different superscript letters within the same row indicate significant differences among dietary groups based on Dunn–Bonferroni pairwise comparisons following the Kruskal–Wallis test, where applicable

### Enzymatic Activities

#### Alkaline protease

Initially, alkaline protease activity was measured as 2.06 U mg protein⁻^1^ in all groups. By the end of the experiment, alkaline protease activity showed a progressive numerical increase with increasing CFP inclusion, from 2.93 ± 0.14 U mg protein⁻^1^ in the control group to 3.29 ± 0.08, 3.89 ± 0.11, and 5.02 ± 0.16 U mg protein⁻^1^ in the CFP1, CFP2, and CFP3 groups, respectively. Dietary treatment significantly affected alkaline protease activity (*p* < 0.05) (Table 11).

**Table 11:** Initial and final digestive enzyme activities in European sea bass fed with different experimental diets (mean ± SE)

	Groups	Initial	Final
Alkaline protease	Control	2.06 ± 0.10	2.93 ± 0.14^a^
	CFP1	2.06 ± 0.10	3.29 ± 0.08^a^
	CFP2	2.06 ± 0.10	3.89 ± 0.11^b^
	CFP3	2.06 ± 0.10	5.02 ± 0.16^c^
Acid protease	Control	1.54 ± 0.16	2.04 ± 0.05^a^
	CFP1	1.54 ± 0.16	2.21 ± 0.20^a^
	CFP2	1.54 ± 0.16	2.72 ± 0.17^ab^
	CFP3	1.54 ± 0.16	3.12 ± 0.17^b^
Amylase	Control	3.89 ± 0.35	5.11 ± 0.24^a^
	CFP1	3.89 ± 0.35	6.08 ± 0.13^ab^
	CFP2	3.89 ± 0.35	6.46 ± 0.28^bc^
	CFP3	3.89 ± 0.35	7.17 ± 0.26^c^
Lipase	Control	2.55 ± 0.15	3.29 ± 0.09^a^
	CFP1	2.55 ± 0.15	3.96 ± 0.13^b^
	CFP2	2.55 ± 0.15	4.25 ± 0.09^b^
	CFP3	2.55 ± 0.15	5.03 ± 0.06^c^

Values are presented as mean ± SE. Initial values are common baseline measurements and were not statistically compared among dietary groups. Final values are tank-level means (*n* = 3 per dietary group). Different superscript letters within the same enzyme row in the final column indicate significant differences according to Tukey’s HSD test following one-way ANOVA (*p* < 0.05)

#### Acid protease

Initially, acid protease activity was 1.54 U mg protein⁻^1^ in all groups. By the end of the trial, acid protease activity increased numerically with CFP inclusion and ranged from 2.04 ± 0.05 U mg protein⁻^1^ in the control group to 3.12 ± 0.17 U mg protein⁻^1^ in the CFP3 group. Dietary treatment had a significant effect on acid protease activity (*p* < 0.05), with the CFP3 group showing higher values than the control and CFP1 groups, while CFP2 remained intermediate (Table 11).

#### Amylase

Initially, amylase activity was 3.89 U mg protein⁻^1^ in all groups. At the end of the experiment, amylase activity increased numerically with CFP inclusion, from 5.11 ± 0.24 U mg protein⁻^1^ in the control group to 6.08 ± 0.13, 6.46 ± 0.28, and 7.17 ± 0.26 U mg protein⁻^1^ in the CFP1, CFP2, and CFP3 groups, respectively. Dietary treatment significantly affected amylase activity (*p* < 0.05), with the highest value observed in the CFP3 group (Table 11).

#### Lipase

Initially, lipase activity was 2.55 U mg protein⁻^1^ in all groups. At the end of the experiment, lipase activity increased numerically with CFP inclusion, from 3.29 ± 0.09 U mg protein⁻^1^ in the control group to 3.96 ± 0.13, 4.25 ± 0.09, and 5.03 ± 0.06 U mg protein⁻^1^ in the CFP1, CFP2, and CFP3 groups, respectively. Dietary treatment significantly affected lipase activity (*p* < 0.05) (Table 11).

### Economic analysis

Numerically lower FCR, diet cost, and ECR values were observed in CFP-supplemented groups, with the lowest calculated ECR in the CFP3 group (Table 12).

**Table 12:** Economic analysis results of sea bass fed with different experimental diets (0%, 25%, 50%, and 100%) for 60 days. ECR: Economic conversion rate, FCR: Feed Conversion Ratio

CFP supplementation level (%)				
	Control	CFP1	CFP2	CFP3
FCR	1.50	1.31	1.26	1.20
Cost of Diet ($)	1.56	1.55	1.54	1.52
ECR	2.35	2.03	1.94	1.83

### Correlation analysis

**Fig. 5:**
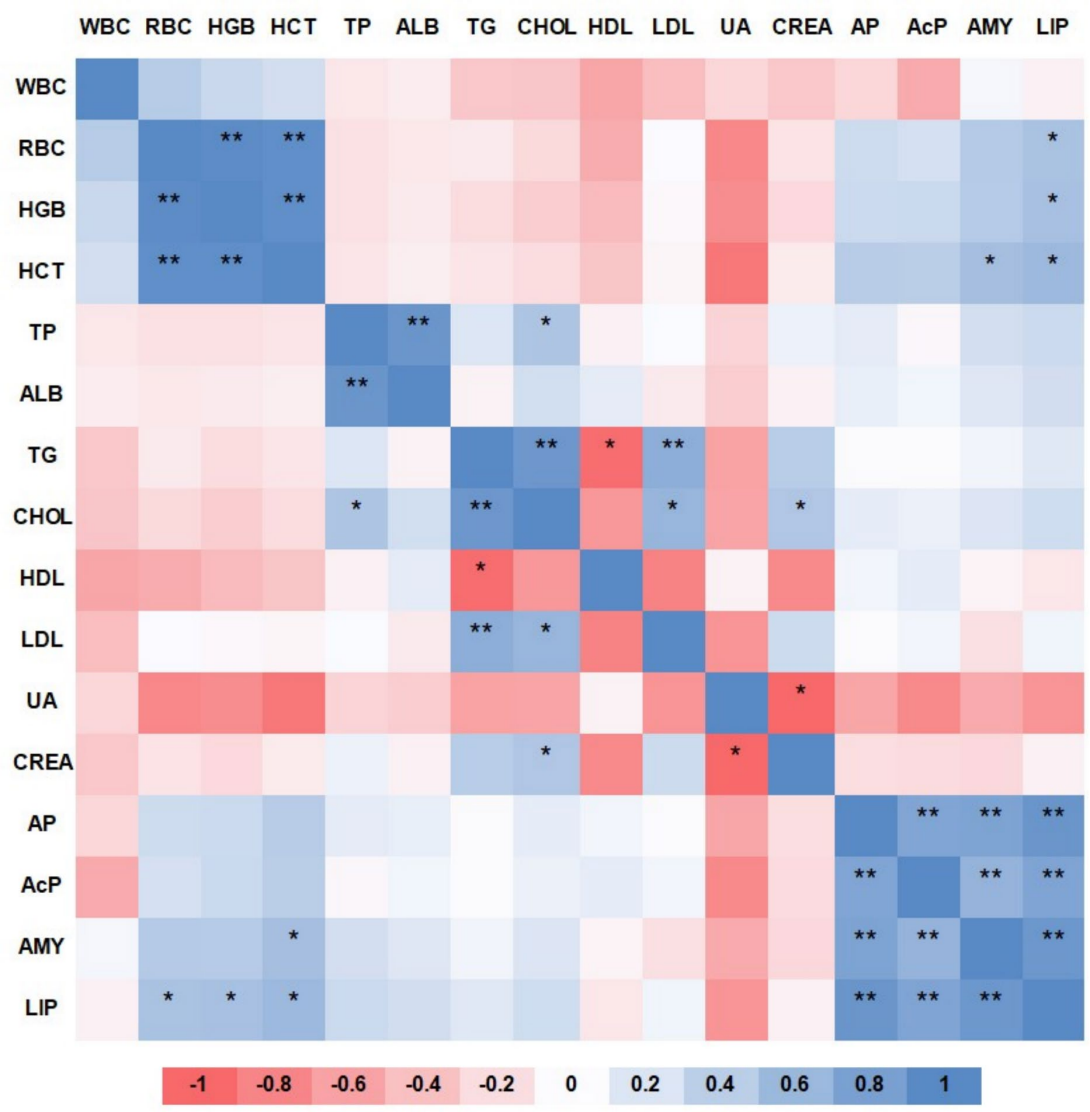
Exploratory heat map showing Pearson correlation coefficients among haematological, serum biochemical, and digestive enzyme variables based on tank-level values. Abbreviations: *WBC* white blood cells, *RBC* red blood cells, *HGB* haemoglobin, *HCT* haematocrit, *TP* total protein, *ALB* albumin, *TG* triglycerides, *CHOL* cholesterol, *HDL* high-density lipoprotein, *LDL* low-density lipoprotein, *UA* uric acid, *CREA* creatinine, *AP* alkaline protease, *AcP *acid protease, *AMY* amylase, *LIP* lipase. Positive correlations are shown in blue and negative correlations in red. * and ** indicate unadjusted two-tailed Pearson significance at *p* < 0.05 and *p* < 0.01, respectively. Correlations were considered exploratory and interpreted descriptively

An exploratory Pearson correlation heat map is shown in Fig. 5; the analysis showed apparent grouping among related haematological, biochemical, and digestive enzyme variables. Erythrocyte-related parameters, particularly RBC, HGB, and HCT, were strongly associated with each other, as expected. Similar positive associations were observed among several lipid-related biochemical variables and among digestive enzyme activities. The heat map also indicated some associations between digestive enzyme activities and haematological parameters.

## Discussion

Fish feed constitutes the largest input item in fish farming and creates high costs, especially for major producers. Fluctuations in the prices of raw materials also lead to cost changes in fish production. For this reason, fish producers prefer low-cost and highly digestible feeds (Boyd and McNevin 2022). In the recent years, alternatives such as DDGS (Distillers Dried Grains with Solubles), HP-DDG (high protein distillers dried grains) and CFP (corn fermented protein), which are a by-product of corn-based ethanol production, have come to the fore (Ray et al. 2022). Within the scope of this study, the effects of CFP added to the feeds of sea bass fry, which are intensively reared in Türkiye, on both aquaculture and physiological parameters were investigated. In addition, the substitution of CFP with corn gluten was examined for the first time in this study. The applied ration is based on the formulas of companies producing commercial fish feed in our country. There are limited studies determining the effects of CFP on enzymatic, histological, digestive physiology and blood parameters. This study is the first to investigate the effects of different levels of CFP instead of corn gluten on growth performance, fillet proximate composition, fatty acid profile, blood parameters, fillet and scale color analysis, nutrient digestibility, economic efficiency, liver and intestinal histology and enzyme parameters of sea bass fry.

Based on the results of this study, the CFP3 group showed numerically higher growth values. This group had the lowest FCR and the highest WG, DGI, and SGR values; however, these differences were not statistically significant. Novriadi et al. (2022) found that the final average weight and thermal growth coefficient of Pacific white shrimp *L. vannamei* fed with 6%, 12% and 18% CFP were significantly higher compared to the control diet. The 18% CFP diet was used partially instead of fish meal and soybean meal and completely instead of corn gluten meal. These findings indicate that the effects of CFP on the growth performance of aquatic organisms should be investigated more comprehensively.

Grayson et al. (2024) reported that gradual replacement of soybean meal with CFP (0%, 25%, 50% and 100%) did not cause any statistically significant difference in WG, SGR, FCR and survival rates in their study on juvenile rainbow trout (*O. mykiss*). Yamamoto et al. (2024) reported that gradual replacement of SBM with CFP at rates of 0%, 25%, 50%, 75% and 100%, respectively, did not cause any significant difference in final weight, feed consumption and survival rate in juvenile channel catfish. These findings indicate that CFP can be used as a potential alternative in aquaculture feeds, but further research is needed to investigate its effects in more depth.

Viscerosomatic index values showed a numerical decrease in the CFP3 group; however, this difference was not statistically significant when tank-level means were used. Hepatosomatic index values were also comparable among dietary groups. Similar to our study, when CFP was used instead of soybean meal in the diets of channel catfish (*Ictalurus punctatus*) fry, HSI values were similar between the control and experimental groups (Yamamoto et al. 2024). These findings suggest that CFP inclusion did not produce clear adverse changes in organ indices or the measured health-related parameters under the present experimental conditions.

In the trial, no significant change was observed in the nutritional composition of sea bass meat when corn gluten was replaced with CFP. Similar to our study, partial and full replacement of soy flour with CFP in juvenile rainbow trout feeds was observed to produce similar results in the nutritional composition of fish meat between groups (Grayson et al. 2024). These findings indicate that plant protein sources can be used without causing a significant change in the nutritional composition of fish fillets.

In the study, the use of CFP instead of corn gluten did not cause a significant change in the fatty acid composition of sea bass. However, slight increases were observed in ΣPUFA, Σn3, and DHA values in the CFP groups. The results indicate that CFP can be used as an alternative protein source while maintaining the fatty acid quality of the fish (Table 4). Similar to our study, Aydın et al. (2017) reported that no significant change was observed in the saturated fatty acid (ΣSFA) levels with the use of DDGS in rainbow trout. Increased levels of linoleic acid (C18:2n-6) and ΣPUFA have been reported.

Imbalanced dietary ingredients or formulations may affect intestinal morphology in the intestinal tissue such as shortening of the mucosal folds and an increase in the width of the submucosa and lamina propria (Krogdahl et al. 2003; Kumar et al. 2020). In contrast, balanced dietary formulations may help maintain intestinal morphology and digestive tissue structure. In previous studies, the inclusion of HP-DDGS up to 50% in sea bass diets led to improvements in mucosal fold parameters (Goda et al. 2019a, 2020). In a CFP study in Atlantic salmon, while the soybean meal ratio decreased and the CFP ratio increased, no significant change in lamina propria was observed at any inclusion level. In addition, villus height was higher at 50% and 100% CFP inclusion, and lamina propria and submucosa measurements showed more favourable values in the 50% CFP group (Hossain et al. 2023). Also, villi height was higher in the 75% CFP group, and lamina propria and submucosa measurements showed more favourable values in the 25% CFP group of the other CFP study in Atlantic salmon (Hong et al. 2025). Similar to the results of Hossain et al. (2023), villus height was highest in the CFP3 group in the present study. In addition, selected intestinal parameters differed among dietary groups in the present study. In the liver, food additives may be associated with hepatocyte vacuolization; in addition, in inflammation reactions, there could be the occurrence of melanomacrophage centers (Seibel et al. 2022). In a study in which fish were fed diets containing 40% and 50% HP-DDGS and soybean meal by 100%, results showed melanomacrophage centers and necrotic areas (Goda et al. 2020). On the other hand, feed prepared with 30% DDGS and 54% fish meal showed no inflammatory reaction in livers in another study (Aydın and Gümüş, 2020). In a study, fish fed with 20% DDGS resulted in bigger hepatocytic vacuoles than the control group (Révész et al. 2019). Similar to that result, hepatocyte diameters were higher in the CFP2 and CFP3 groups in the present study. Moreover, in the 40% DDGS group, livers showed decreasing fat vacuolation and some necrotic hepatocytes at the end of the study (Révész et al. 2019). In contrast to Révész et al. (2019) and Goda et al. (2020), no clear inflammatory changes were observed in the CFP groups in the present study. Therefore, the increased hepatocyte diameter observed in CFP2 and CFP3 may reflect changes in hepatocyte vacuolization or cellular morphology; however, this interpretation should be made cautiously because specific lipid staining was not performed. This interpretation is also consistent with the absence of significant differences in hepatosomatic index among dietary groups.

Hematological parameters are generally accepted as an effective indicator for detecting stress or disease conditions in fish (Fazio 2019). Especially changes in red and white blood cell count, hematocrit and hemoglobin values ​​provide important information for monitoring organ health (Witeska et al. 2023). In this study, the results (Table 6) revealed that CFP groups did not show any significant difference in hematological parameters (WBC, RBC, Hb and Hct) at the end of 60 days when compared with the control group. In a study conducted by Goda et al. (2019b), an increase was detected in WBC, RBC, Hb and Hct values ​​of sea bass fry fed a diet containing phytase and HP-DDG. In addition, Goda et al. (2019a) reported an increase in WBC, RBC and Hb values ​​in sea bass fry fed with HP-DDG in another study. In conclusion, in this study, CFP groups did not show any significant change in hematological parameters compared to the control group, which is different from the results reported in different studies. This situation indicates that further research should be done on whether the diet used influences the hematological parameters of the fish.

Serum protein, albumin, and globulin levels have been suggested to be associated with a stronger innate immune response in fish (Wiegertjes et al. 1996). In this study, TP, ALB and GLO levels of the CFP supplemented groups were found to be similar compared to the control group. Goda et al. (2019a) reported that total serum protein, albumin and globulin levels increased in sea bass fed with HP-DDG. In another study by Goda et al. (2019b), it was observed that total protein, albumin and globulin levels increased with the addition of HP-DDG and phytase to the diet of sea bass fry. In addition, in another study by Goda et al. (2020), it was found that total serum protein, albumin and globulin levels increased in sea bass fry fed diets containing increased HP-DDG levels and phytase supplementation. These findings reveal the effects of dietary components on serum protein levels of fish and emphasize the importance of further research on this subject. In fish fed CFP diets, no significant changes were observed in serum TG, CHOL, HDL, LDL and VLDL levels compared to the control group. In a study by Goda et al. (2019a), it was found that CHOL levels increased significantly with increasing HP-DDG levels in sea bass fish. In another study by Goda et al. (2019b), it was observed that CHOL levels in sea bass fry increased significantly with increasing levels of diets containing HPDDG and phytase. In addition, in another study by Goda et al. (2020), it was found that both TG and CHOL levels increased in sea bass fry fed diets containing increased HP-DDG levels and phytase supplements. This suggests that dietary components may have different effects on the serum parameters of fish. The physiological roles of urea, uric acid and creatinine have not yet been fully elucidated (Yılmaz et al. 2012). However, these compounds are used as useful indicators in the evaluation of important parameters such as general status of gill and kidney health, feed efficiency and amino acid (arginine) requirements in fish (Campbell 2004; Tulli et al. 2007; Tibaldi et al. 1995). In this study, CFP did not change serum uric acid and creatinine levels. Consequently, the lack of change in serum uric acid and creatinine levels may indicate that the metabolic balance of the fish is not disturbed, that treatment or nutritional interventions are ineffective or that environmental conditions are stable.

In our study, replacing corn gluten with CFP in the diets resulted in increased L*, a*, and b* values of the feeds. The highest values for all color parameters were observed particularly in the CFP2 group. In contrast, no significant changes were observed in the scale and fillet color among the experimental groups. In the study of Aydın et al. (2017), L* values were found similar between the experimental feeds, while a* and b* values differed between the control and experimental groups. The high b* value of DDGS was also reflected in the feeds containing DDGS. While no significant difference was observed between the groups in the skin and fillet color measurements taken at the end of the experiment, a difference was found between the DDGS10 group and the control group in terms of L* value in the dorsal and caudal regions. Yıldırım et al. (2024) study, it was determined that the use of DDGS instead of soybean meal was effective on rainbow trout meat color. With the increase in DDGS level, the L* value increased, the a* value decreased, but the b* value did not change. As a result, it is seen that the addition of DDGS and CFP to the diets can affect the color of fish fillets, but this effect varies depending on the ingredients and diet formulations used.

In our study, dry matter, protein and fat apparent digestibility coefficients were found to be similar. In the study by Grayson et al. (2024), lipid apparent digestibility was found to be similar in diets in which soybean meal was gradually replaced with CFP in juvenile rainbow trout, but protein apparent digestibility was approximately 3% higher in the diet group containing 50% CFP than in the control group. In a different study, when CFP was used instead of soybean meal in diets of juvenile salmon, protein ADC of the SBM-containing diet was found to be higher than the CFP-containing diet and reference diets. Lipid ADC was found to be similar to the CFP-containing diet (Yamamoto et al. 2024). Since there is limited data on the effects of partial replacement of corn gluten with different CFP inclusion levels on the digestibility of aquatic organisms, further research is required on this subject.

Digestive enzyme activity is widely used as an indicator of digestive capacity and of the physiological response of fish to dietary composition. In the present study, dietary CFP inclusion was associated with significant differences in alkaline protease, acid protease, amylase, and lipase activities, with the highest values generally observed in the CFP3 group. Acid protease activity mainly reflects the action of gastric proteolytic enzymes under acidic conditions, whereas alkaline protease activity is more closely related to intestinal proteases involved in protein digestion. In addition, amylase and lipase play important roles in the hydrolysis of carbohydrates and lipids, respectively. Taken together, these findings suggest that increasing CFP inclusion may have influenced digestive physiology in juvenile European sea bass.

Comparable responses were reported in our previous study with rainbow trout juveniles fed DDGS-containing diets, where digestive enzyme activities also increased with dietary inclusion level. In the present study, one possible explanation for the higher enzyme activities observed in CFP-fed groups is the different nutritional composition of CFP relative to the control diet, including its protein and carbohydrate fractions. The increase in amylase activity may also be related to the starch-containing binders used during diet preparation. However, these interpretations should be considered cautiously, and the observed increases in enzyme activity should not be taken as direct evidence of improved growth or feed utilization, since growth-related variables were not significantly affected under the present experimental conditions.

In the present study, replacing corn gluten with CFP did not significantly affect growth performance, feed utilization, haematological or serum biochemical parameters, apparent digestibility, or the major whole-body fatty acid groups under the conditions of the 60-day trial. However, whole-body lipid content, digestive enzyme activities, and selected intestinal histomorphometric parameters were affected by dietary treatment. Although the CFP3 group showed numerically higher growth values and lower ECR, these changes should be interpreted as numerical trends rather than evidence of improved feed efficiency. Overall, the results suggest that CFP can replace the corn-gluten fraction at the tested inclusion levels without clear adverse effects on the measured growth and health-related parameters, while the observed changes in lipid content, intestinal morphology, and enzyme activities require further investigation in longer-term trials and under different culture conditions.

## Conclusion

Under the conditions of this 60-day trial, corn fermented protein (CFP) replaced the corn-gluten fraction at the tested inclusion levels without significantly affecting growth or feed utilization; group means for WG, DGI, FCR, and SGR were statistically similar (*p* > 0.05). Digestive enzyme activities and selected intestinal histomorphometric parameters were affected, although these changes were not accompanied by significant differences in apparent digestibility. CFP inclusion did not significantly alter the whole-body fatty acid profile of sea bass; although some numerical increases were observed in PUFA-related fatty acid variables, these differences were not statistically significant. Overall, CFP may be a practical option for replacing corn gluten in juvenile European sea bass diets under the tested conditions, with potential effects on digestive physiology that require confirmation in longer trials, additional life stages, and different economic conditions.

## Data Availability

The datasets generated and analyzed during the current study are available from the corresponding author upon reasonable request.
